# Prevalence and associations of axillary web syndrome in Asian women after breast cancer surgery undergoing a community-based cancer rehabilitation program

**DOI:** 10.1186/s12885-021-08762-z

**Published:** 2021-09-14

**Authors:** Matthew Rong Jie Tay, Chin Jung Wong, Hui Zhen Aw

**Affiliations:** 1 Department of Rehabilitation Medicine, Tan Tock Seng Hospital, 11 Jalan Tan Tock Seng, Singapore, 308433 Singapore; 2 Singapore Cancer Society Rehabilitation Center, Singapore Cancer Society, Singapore, Singapore

**Keywords:** Cording, Mondor’s disease, Breast surgery, Axillary lymphadenectomy, Lymph node dissection, Post-operative morbidity

## Abstract

**Background:**

Patients who have breast cancer surgery are at risk of axillary web syndrome (AWS), an under-recognized postsurgical complication which can result in shoulder morbidity and functional impairment. Emerging studies have indicated that AWS may persist beyond the first few months after surgery, although few studies have assessed the prevalence and association of AWS beyond a year after diagnosis. Therefore, the aim of this study was to investigate the prevalence and associations for AWS in post-operative breast cancer patients up to 3 years after surgery.

**Methods:**

This cross sectional observational study was conducted at a community-based cancer rehabilitation center. Patients were evaluated for the presence of AWS via physical examination. Disease-related data was obtained from clinical review and medical records. Descriptive statistics were utilized to illustrate patient demographics and clinical characteristics. Logistic regression analyses were used to determine associations of AWS.

**Results:**

There were 111 Asian women who were recruited, who had undergone breast surgery and were referred to a national outpatient rehabilitation center. The prevalence of AWS in this population was 28.9%. In the multivariate regression model, significant factors were age < 50 years (OR = 3.51; 95% CI = 1.12–11.0; *p* = 0.031) and ALND (OR = 6.54; 95% CI = 1.36–31.3; *p* = 0.019). There was reduced shoulder flexion ROM (*p* < 0.001) in patients with AWS compared to patients without AWS.

**Conclusions:**

A high prevalence of AWS was reported in breast cancer survivors even at 3 years after breast surgery. Our findings highlight the need to identify breast cancer survivors with AWS even in the survivorship phase, and develop strategies to raise awareness and minimize functional impairment in these patients.

## Background

Breast cancer surgery is the main treatment of breast cancer which is often combined with either sentinel lymph node biopsy (SLNB) or axillary lymph node dissection (ALND) for prognostication and therapeutic decision making. However, such axillary approaches may result in postoperative morbidity, such as pain, lymphedema, shoulder dysfunction and postoperative infection [1].

Axillary web syndrome (AWS) is a common, but under-recognized postsurgical complication in patients following breast surgery. The diagnosis is clinical, and is characterized by the presence of cording, where one or more visible or palpable cords are present in the axilla, which can extend down to the medial ipsilateral arm, frequently to the antecubital space and occasionally to the base of the thumb [2]. This can result in pain, limited shoulder range of motion, postural impairment, numbness, functional impairment of the shoulder joint, psychological problems and impaired quality of life [3, 4]. Although pain and range of motion limitation are the most commonly reported complications [5], AWS can result in disability of the shoulder and even impair return to work [6, 7]. AWS is believed to be associated with a lymphatic origin [8–11], and evaluation should exclude Mondor’s disease, a condition which is caused by superficial thrombophlebitis, and may present similarly with palpable cord-like induration over the mid-upper arm [12–16].

The incidence of AWS has been reported to range widely from 4 to 85%, depending on the duration and frequency of postoperative follow-up and the type of axillary surgery, with the majority of studies reporting AWS occurrence within 8 weeks of breast surgery [5]. More invasive breast cancer surgery is believed to be linked with AWS occurrence, with de Sire et al. reporting ALND to be a significant risk factor for AWS [17]. Other risk factors for AWS development include younger age [18, 19], lower body mass index [20, 21], healing complications [22] and adjunctive therapies [17]. A possible link between breast cancer related lymphedema and AWS has also been hypothesized, with some studies suggesting that AWS may increase the risk of developing lymphedema [17, 23].

This condition is believed to occur soon after axillary surgery and is thought to be self-limiting with most patients experiencing spontaneous resolution within 3 months [24, 25]. However, emerging data have demonstrated that AWS may persist longer than 12 weeks post surgery, and may even be diagnosed on follow-up after 18 months [23, 26]. AWS is increasing being diagnosed in the postoperative rehabilitative setting, and it is important to understand the prevalence of AWS, so as to develop survivorship guidelines for patients with breast cancer in order to enable more effective social reintegration of patients and to limit surgical sequelae [27–30]. The aim of this study was to investigate the prevalence and associations for AWS in post-operative breast cancer patients up to 3 years after surgery in an outpatient community cancer rehabilitation program.

## Methods

### Participants

This is a cross sectional study of women of Asian ethnicity who had undergone breast surgery who presented at an outpatient-based cancer rehabilitation clinic for an initial evaluation between September 2017 to August 2019. This is the only outpatient multi-disciplinary community cancer rehabilitation program available in Singapore currently, and these breast cancer survivors were referred from clinical specialists or primary care physicians from any local healthcare institutions after they had completed their acute oncological treatment [31]. It provides comprehensive outpatient rehabilitation services with a physician-led multidisciplinary team, which includes physiotherapists, nutritionists, medical social workers and psychologists. Patients were assessed by a rehabilitation physician and physiotherapist during their initial evaluation.

The clinical and pathological data were collected through chart reviews and patient interview. Data collected included age, ethnicity, stage of breast cancer and the type of breast cancer treatment. Breast cancer surgery was categorized as either breast-conserving surgery or simple mastectomy with or without reconstruction. Lymph node surgery was categorized as either SNLB or ALND.

### Inclusion and exclusion criteria

Eligibility included women ≥21 years old who had undergone breast cancer surgery with a postoperative period of up to 3 years, with either a SLNB or an ALND. Women were excluded if they had attended the program < 1 month from surgery, had other active malignant tumors, presence of systemic metastases, previous surgery for breast cancer, synchronous bilateral breast cancer or a previous history of shoulder surgery, shoulder dysfunction or upper extremity deep vein thrombosis.

This clinical study was performed in accordance with the principles of the Declaration of Helsinki. This audit study was approved by the local institutional review board, Agency for Integrated Care (2019–009).

### Patient evaluation

The initial evaluation involved assessing for the presence of AWS, pain, shoulder range of motion and lymphedema.

Diagnosis of AWS was made through physical examination by a physiotherapist and confirmed by the physician, and defined as having palpable or visible axillary cords of tissue anywhere from the axilla, which may or may not extend to the ipsilateral arm [2].

We also assessed the presence of pain and restricted shoulder range of motion. The range of motion (ROM) was measured for forward flexion, abduction and external rotation of both arms through the use of a goniometer, while the patient was in a sitting position. An ROM difference of 10 or more degrees between the ipsilateral and contralateral extremity in either shoulder flexion or abduction indicated reduced ROM [32, 33]. The presence of pain was defined as a Visual Analog Pain scale of 3 or more [34, 35]. Lymphedema was diagnosed by a physician, and further evaluation was performed by a trained lymphedema physiotherapist. Arm-circumference measurements or self-reported symptoms were used to diagnose lymphedema. Patients who wore compression sleeves removed them 1 h before arm measurements were obtained. An arm circumference of more than 2 cm between affected and non-affected side was indicative of lymphedema [36, 37]. Lymphedema symptoms elicited included whether or not a participant had noticed that her hand, upper or lower arm on the side of the cancer was larger, puffier and/or swollen [36].

### Statistical analysis

Descriptive statistics were used to illustrate patient demographics and clinical characteristics. Logistic regression analyses were utilized for the univariate and multivariable analyses of the associations with AWS.

Covariates in the unadjusted analyses were then included in the subsequent multivariable regression model.

A *p*-value < 0.05 was considered statistically significant for a two-tailed test. Statistical analyses were performed using the Statistical Package for the Social Sciences Version 25.0 (IBM Corp., Armonk, NY, USA).

## Results

There were 111 Asian women recruited, with 32 (28.9%) diagnosed with AWS. The majority of patients with AWS presented at 1 or 2 years post surgery (84.8%), though there were 12 patients (15.2%) who presented at 3 years post surgery. There were 25 (31.6%) and 22 (27.8%) patients who also had arm pain and lymphedema respectively (Table 1).

**Table 1:** Characteristics of the study population (*N* = 111)

	Axillary web syndrome		Total
	No (*N* = 79)	Yes (*N* = 32)	*N* = 111
**Characteristics**			
**Age at surgery,*n*(%)**			
**- < 50 years**	12 (15.2)	12 (37.5)	24 (21.6)
**- > =50 years**	67 (84.8)	20 (62.5)	87 (78.4)
**Ethnicity,*n*(%)**			
**- Chinese**	73 (92.4)	27 (84.4)	100 (90.1)
**- Malay**	5 (6.3)	4 (12.5)	9 (8.1)
**- Indian**	1 (1.3)	1 (3.1)	2 (1.8)
**Duration post surgery,*n*(%)**			
**- 1 year**	32 (40.5)	16 (50.0)	48 (43.2)
**- 2 years**	35 (44.3)	14 (43.8)	49 (44.1)
**- 3 years**	12 (15.2)	2 (6.3)	14 (12.6)
**Affected upper extremity,*n*(%)**			
**- Left**	31 (39.2)	15 (46.9)	46 (41.4)
**- Right**	48 (60.8)	17 (53.1)	65 (58.6)
**Type of breast cancer surgery,*n*(%)**			
**- Breast conserving surgery**	23 (29.1)	9 (28.1)	32 (28.8)
**- Simple mastectomy**	47 (59.5)	17 (53.1)	64 (57.7)
**- Simple mastectomy with reconstruction**	9 (11.4)	6 (18.8)	15 (13.5)
**Axillary procedure,*n*(%)**			
**- SLNB**	43 (54.4)	7 (21.9)	50 (45.0)
**- ALND**	36 (45.6)	25 (78.1)	61 (55.0)
**Adjunctive therapy,*n*(%)**			
**- Chemotherapy**	58 (73.4)	25 (78.1)	83 (74.8)
**- Radiotherapy**	47 (59.5)	23 (71.9)	70 (63.1)
**Stage of cancer,*n*(%)**			
**- I**	37 (46.8)	8 (25.0)	45 (40.5)
**- II**	38 (48.1)	20 (62.5)	58 (52.3)
**- III**	4 (5.1)	4 (12.5)	8 (7.2)
**Presence of arm pain,*n*(%)**	25 (31.6)	18 (56.3)	39 (35.1)
**Presence of lymphedema,*n*(%)**	22 (27.8)	11 (34.4)	33 (29.7)
**Flexion active ROM (degrees), mean (SD)**	159.7 (5.21)	143.8 (23.2)	152.8 (17.5)
**Abduction active ROM (degrees), mean (SD)**	146.9 (25.6)	133.9 (30.0)	141.2 (28.1)
**External rotation active ROM (degrees), mean (SD)**	53.0 (8.50)	50.9 (7.11)	52.1 (7.91)

*SLNB* sentinel lymph node biopsy, *ALND* axillary lymph node dissection, *ROM* range of motion, *SD* standard deviation

The majority of patients with AWS had simple mastectomy without reconstruction (59.5%), and also received adjunctive chemotherapy (73.4%) and radiotherapy (59.5%). Most patients either had stage I (46.8%) or II (48.1%) breast cancer. Among the patients who underwent ALND, there were 25 patients (41.0%) with AWS (Table 1).

More patients with AWS had limitation in shoulder flexion (*p* < 0.001) compared to those without AWS. However, there were no difference in shoulder abduction (*p* = 0.078), shoulder external rotation (*p* = 0.300), pain (*p* = 0.228) or lymphedema (*p* = 0.496) between both groups (Table 1).

In univariate analysis, significant associations with AWS were age < 50 years (Odds ratio [OR] = 2.93; 95% confidence interval [CI] = 1.13–7.59; *p* = 0.027) and ALND (OR = 4.27; 95% CI = 1.65–11.0; *p* = 0.003) (Table 2).

**Table 2:** Univariate and multivariate analysis of associations with axillary web syndrome

Characteristics	Univariate analysis		Multivariate analysis	
	Odds ratio (95% CI)	*p*-value	Odds ratio (95% CI)	*p*-value
Age at surgery				
**- > =50 years**	1.0		1.0	
**- < 50 years**	2.93 (1.13–7.59)	0.027	3.51 (1.12–11.0)	0.031
**Ethnicity**				
**- Chinese**	1.0		1.0	
**- Malay**	2.16 (0.540–8.66)	0.216	2.27 (0.421–12.3)	0.340
**- Indian**	2.70 (0.163–44.8)	0.487	0.685 (0.028–16.9)	0.817
**Duration post surgery**				
**- 1 year**	1.0		1.0	
**- 2 years**	0.800 (0.338–1.90)	0.612	1.30 (0.436–3.90)	0.636
**- 3 years**	0.333 (0.066–1.67)	0.182	0.295 (0.043–2.03)	0.215
**Affected upper extremity**				
**- Left**	1.0		1.0	
**- Right**	0.732 (0.320–1.68)	0.460	0.985 (0.340–2.85)	0.977
**Type of breast cancer surgery**				
**- Breast conserving surgery**	1.0		1.0	
**- Simple mastectomy**	0.924 (0.358–2.39)	0.871	0.359 (0.103–1.25)	0.106
**- Simple mastectomy with reconstruction**	1.70 (0.470–6.18)	0.418	0.759 (0.148–3.89)	0.740
**Axillary procedure**				
**- SLNB**	1.0		1.0	
**- ALND**	4.27 (1.65–11.0)	0.003	6.54 (1.36–31.3)	0.019
**Adjunctive therapy**				
**- Chemotherapy**	1.29 (0.488–3.43)	0.606	0.498 (0.139–1.79)	0.285
**- Radiotherapy**	1.74 (0.713–4.25)	0.224	1.01 (0.304–3.32)	0.993
**Stage of cancer**				
**- I**	1.0		1.0	
**- II**	2.43 (0.954–6.21)	0.063	1.13 (0.282–4.52)	0.864
**- III**	4.63 (0.950–22.5)	0.058	2.68 (0.312–23.1)	0.369
**Presence of pain**	1.68 (0.722–3.91)	0.228	1.59 (0.561–4.51)	0.383
**Presence of lymphedema**	1.36 (0.563–3.27)	0.496	0.849 (0.253–2.85)	0.791

*SLNB* sentinel lymph node biopsy, *ALND* axillary lymph node dissection

In the multivariate regression model, significant factors for AWS were age < 50 years (OR = 3.51; 95% CI = 1.12–11.0; *p* = 0.031) and ALND (OR = 6.54; 95% CI = 1.36–31.3; *p* = 0.019) (Table 2).

## Discussion

The major finding is a prevalence of 28.9% of AWS in our study cohort, diagnosed objectively via physical examination. A prospective cohort study by Koehler et al. of 36 patients found a cumulative prevalence of 50% of AWS at 18 months following breast cancer surgery [26], while another prospective cohort by O′ Toole et al. found a cumulative incidence of 31.5% at 24 months post-operatively [23]. This study cohort, which had patients who were recruited up to 3 years after breast cancer surgery, extends the aforementioned studies, by demonstrating that AWS can still present as a late post-operative breast surgery complication. Moreover, it is possible that our prevalence in our study population may had been an underestimate, as patients assessed at the initial evaluation may had experienced prior AWS that had since resolved.

The late presentation of AWS may also be due to under-diagnosis by patients and non-rehabilitative providers due to unfamiliarity with this condition, resulting in delayed treatment [38]. Our findings has implications for rehabilitation providers, given that reduced shoulder flexion and abduction ROM were also reported in our study cohort, which is consistent with the findings in AWS [5]. This highlights the need of healthcare professionals and patients to be educated on the recognition of AWS, and to initiate early intervention strategies. This is especially pertinent due to the potential detrimental effects of reduced ROM and pain on upper extremity function and arm volume elevation [23, 26, 39].

We also found that ALND and a younger age were significantly associated with AWS, with an odds ratio of 3.51 and 6.54 respectively. AWS was found to be present in 41.0% of women who underwent ALND, which is similar to studies performed in patients with breast cancer [5, 22]. The increased risk of AWS with ALND may be due to a more invasive surgical intervention increasing the risk of interruption of axillary lymphatics or thrombosed lymphatic vessels, although the exact patho-mechanisms remains yet unknown [5].

Several other studies have also reported a younger age as a risk factor for AWS [5], with Brunelle et al., for example, found an age less than 55 years to be a significant risk factor for AWS, which corroborates our findings [39]. While some studies have suggested that lymphedema may be associated with AWS, other publications have found no relevant relationship, with our study also finding no such significant association between these 2 variables [5, 40]. Additionally, while pain associated with AWS has been reported in patients in the early postoperative period, we did not find this to be more prevalent in patients with AWS, which is in accordance with studies investigating patients in the post-acute follow-up period [23, 26].

### Limitations

There are several limitations to be considered in this study. First, we used a convenience sample of patients presenting to a rehabilitation center, which may result in a higher reported prevalence of AWS compared to a outpatient oncology clinic setting. Second, the study had a small sample size and was cross-sectional in nature. A larger, prospective study will be needed to confirm our findings, and also define the evolution of AWS and response to rehabilitative interventions over time [41]. Third, we did not investigate other known associations with AWS, including body mass index or presence of postoperative complications (e.g. hematoma, intercostobrachial nerve injuries) [42]. We also did not quantify whether the extent of lymph node dissection were risk factors for AWS. Fourth, data on impairments in functional tasks were not available, and further studies on impairment outcomes and quality of life scores are needed to determine the clinical impact of AWS on breast cancer survivorship [21].

## Conclusion

In conclusion, the prevalence of AWS after breast cancer surgery is high even after the acute postoperative period. Given that AWS is manifestly treatable with interventions such as therapeutic exercise and manual therapy, breast cancer survivors and care providers should be educated about this condition so that early referral and management can reduce long term morbidity in patients. Further studies on a larger population of breast cancer survivors, with objective measurement of shoulder kinematics are needed to confirm our findings, and delineate the biomechanical effects of AWS on shoulder motion loss.

## Data Availability

The datasets used and/or analyzed during the current study are available from the corresponding author on reasonable request.

## Abbreviations

AWS: Axillary web syndrome
SLNB: Sentinel lymph node biopsy
ALND: Axillary lymph node dissection
ROM: Range of motion
SD: Standard deviation
CI: Confidence interval

## References

[1] Olsson Möller U, Beck I, Rydén L, Malmström M (2019). A comprehensive approach to rehabilitation interventions following breast cancer treatment - a systematic review of systematic reviews. BMC Cancer.

[2] Moskovitz AH, Anderson BO, Yeung RS, Byrd DR, Lawton TJ, Moe RE (2001). Axillary web syndrome after axillary dissection. Am J Surg.

[3] Piper M, Guajardo I, Denkler K, Sbitany H (2016). Axillary web syndrome: current understanding and new directions for treatment. Ann Plast Surg.

[4] Paolucci T, Bernetti A, Paoloni M, Capobianco SV, Bai AV, Lai C, Pierro L, Rotundi M, Damiani C, Santilli V, Agostini F, Mangone M (2019). Therapeutic Alliance in a single versus group rehabilitative setting after breast Cancer surgery: psychological profile and performance rehabilitation. Biores Open Access.

[5] Yeung WM, McPhail SM, Kuys SS (2015). A systematic review of axillary web syndrome (AWS). J Cancer Surviv.

[6] Lattanzi JB, Zimmerman A, Marshall LM (2012). Case report of axillary web syndrome. Rehabil Oncol.

[7] Fourie WJ, Robb KA (2009). Physiotherapy management of axillary web syndrome following breast cancer treatment: discussing the use of soft tissue techniques. Physiotherapy..

[8] Rashtak S, Gamble GL, Gibson LE, Pittelkow MR (2012). From furuncle to axillary web syndrome: shedding light on histopathology and pathogenesis. Dermatology..

[9] Tilley A, Thomas-Maclean R, Kwan W (2009). Lymphatic cording or axillary web syndrome after breast cancer surgery. Can J Surg.

[10] Johansson K, Chong H, Ciornei CD, Brorson H, Mortimer PS (2020). Axillary web syndrome: evidence for lymphatic origin with thrombosis. Lymphat Res Biol.

[11] Rockson SG (2020). The beguiling histopathology of the axillary web syndrome. Lymphat Res Biol.

[12] Leduc O, Fumière E, Banse S, Vandervorst C, Clément A, Parijs T, Wilputte F, Maquerlot F, Ezquer Echandia M, Tinlot A, Leduc A (2014). Identification and description of the axillary web syndrome (AWS) by clinical signs. MRI US Imaging Lymphol.

[13] Cho Y, Do J, Jung S, Kwon O, Jeon JY (2016). Effects of a physical therapy program combined with manual lymphatic drainage on shoulder function, quality of life, lymphedema incidence, and pain in breast cancer patients with axillary web syndrome following axillary dissection. Support Care Cancer.

[14] de Sire A, Invernizzi M, Lippi L, Cisari C, Özçakar L, Franchignoni F (2020). Blurred lines between axillary web syndrome and Mondor's disease after breast cancer surgery: a case report. Ann Phys Rehabil Med.

[15] Amano M, Shimizu T (2018). Mondor's disease: a review of the literature. Intern Med.

[16] Pasta V, D'Orazi V, Sottile D, Del Vecchio L, Panunzi A, Urciuoli P (2015). Breast Mondor's disease: diagnosis and management of six new cases of this underestimated pathology. Phlebology..

[17] de Sire A, Losco L, Cisari C, Gennari A, Boldorini R, Fusco N, Cigna E, Invernizzi M (2020). Axillary web syndrome in women after breast cancer surgery referred to an oncological rehabilitation unit: which are the main risk factors? A retrospective case-control study. Eur Rev Med Pharmacol Sci.

[18] Fukushima KF, Carmo LA, Borinelli AC, Ferreira CW (2015). Frequency and associated factors of axillary web syndrome in women who had undergone breast cancer surgery: a transversal and retrospective study. Springerplus..

[19] Koehler LA, Blaes AH, Haddad TC, Hunter DW, Hirsch AT, Ludewig PM (2015). Movement, function, pain, and postoperative edema in axillary web syndrome. Phys Ther.

[20] Torres Lacomba M, Mayoral Del Moral O, Coperias Zazo JL, Yuste Sánchez MJ, Ferrandez JC, Zapico GA (2009). Axillary web syndrome after axillary dissection in breast cancer: a prospective study. Breast Cancer Res Treat.

[21] Baggi F, Nevola Teixeira LF, Gandini S, Simoncini MC, Bonacossa E, Sandrin F, Sciotto Marotta M, Lanni G, Dadda P, Colpani D, Luini A (2018). Axillary web syndrome assessment using a self-assessment questionnaire: a prospective cohort study. Support Care Cancer.

[22] Bergmann A, Mendes VV, de Almeida DR (2012). Do Amaral E Silva B, da Costa Leite Ferreira MG, Fabro EA. incidence and risk factors for axillary web syndrome after breast cancer surgery. Breast Cancer Res Treat.

[23] O'Toole J, Miller CL, Specht MC, Skolny MN, Jammallo LS, Horick N (2013). Cording following treatment for breast cancer. Breast Cancer Res Treat.

[24] Leidenius M, Leppänen E, Krogerus L, von Smitten K (2003). Motion restriction and axillary web syndrome after sentinel node biopsy and axillary clearance in breast cancer. Am J Surg.

[25] Aydogan F, Belli AK, Bağhaki S, Karabulut K, Tahan G, Uras C (2008). Axillary web syndrome after sentinel node biopsy. Breast Care (Basel).

[26] Koehler LA, Hunter DW, Blaes AH, Haddad TC (2018). Function, shoulder motion, pain, and lymphedema in breast Cancer with and without axillary web syndrome: an 18-month follow-up. Phys Ther.

[27] Harris SR (2018). Axillary Web Syndrome in Breast Cancer: A Prevalent But Under-Recognized Postoperative Complication. Breast Care (Basel).

[28] Luz CMD, Deitos J, Siqueira TC, Palú M, Heck APF (2017). Management of Axillary web Syndrome after breast Cancer: evidence-based practice. Rev Bras Ginecol Obstet.

[29] Thompson Buum HA, Koehler L, Tuttle TM (2017). Venturing out on a limb: axillary web syndrome. Am J Med.

[30] Dinas K, Kalder M, Zepiridis L, Mavromatidis G, Pratilas G (2019). Axillary web syndrome: incidence, pathogenesis, and management. Curr Probl Cancer.

[31] Wong CJ, Tay MRJ, Aw HZ (2021). Prevalence and risk factors of adhesive capsulitis in Asian breast Cancer patients undergoing an outpatient community Cancer rehabilitation program. Arch Phys Med Rehabil.

[32] Verbelen H, Gebruers N, Eeckhout FM, Verlinden K, Tjalma W (2014). Shoulder and arm morbidity in sentinel node-negative breast cancer patients: a systematic review. Breast Cancer Res Treat.

[33] Kuehn T, Klauss W, Darsow M, Regele S, Flock F, Maiterth C, Dahlbender R, Wendt I, Kreienberg R (2000). Long-term morbidity following axillary dissection in breast cancer patients--clinical assessment, significance for life quality and the impact of demographic, oncologic and therapeutic factors. Breast Cancer Res Treat.

[34] Janz NK, Mujahid M, Chung LK, Lantz PM, Hawley ST, Morrow M, Schwartz K, Katz SJ (2007). Symptom experience and quality of life of women following breast cancer treatment. J Women's Health (Larchmt).

[35] Tengrup I, Tennvall-Nittby L, Christiansson I, Laurin M (2000). Arm morbidity after breast-conserving therapy for breast cancer. Acta Oncol.

[36] Ahmed RL, Thomas W, Yee D, Schmitz KH (2006). Randomized controlled trial of weight training and lymphedema in breast cancer survivors [published correction appears in J Clin Oncol. 2006;24:3716]. J Clin Oncol.

[37] Harris SR, Hugi MR, Olivotto IA, Levine M (2001). Steering Committee for Clinical Practice Guidelines for the care and treatment of breast Cancer. Clinical practice guidelines for the care and treatment of breast cancer: 11. Lymphedema. CMAJ..

[38] Koehler LA, Haddad TC, Hunter DW, Tuttle TM (2018). Axillary web syndrome following breast cancer surgery: symptoms, complications, and management strategies. Breast Cancer (Dove Med Press).

[39] Brunelle CL, Roberts SA, Shui AM, Gillespie TC, Daniell KM, Naoum GE, Taghian A (2020). Patients who report cording after breast cancer surgery are at higher risk of lymphedema: results from a large prospective screening cohort. J Surg Oncol.

[40] Ryans K, Davies CC, Gaw G, Lambe C, Henninge M, VanHoose L (2020). Incidence and predictors of axillary web syndrome and its association with lymphedema in women following breast cancer treatment: a retrospective study. Support Care Cancer.

[41] Beurskens CH, van Uden CJ, Strobbe LJ, Oostendorp RA, Wobbes T (2007). The efficacy of physiotherapy upon shoulder function following axillary dissection in breast cancer, a randomized controlled study. BMC Cancer.

[42] Ramírez-Parada K, Garay-Acevedo D, Mella-Abarca W, Petric-Guajardo M, Sánchez-Rojel C, McNeely ML, Leao-Ribeiro I (2020). Axillary web syndrome among Chilean women with breast cancer: incidence and possible predisposing factors. Support Care Cancer.

